# Homeodomain-Interacting Protein Kinase (HPK-1) regulates stress responses and ageing in *C. elegans*

**DOI:** 10.1038/srep19582

**Published:** 2016-01-21

**Authors:** Slavica Berber, Mallory Wood, Estelle Llamosas, Priya Thaivalappil, Karen Lee, Bing Mana Liao, Yee Lian Chew, Aaron Rhodes, Duygu Yucel, Merlin Crossley, Hannah R Nicholas

**Affiliations:** 1 School of Molecular Bioscience, University of Sydney, Sydney, Australia; 2 School of Biotechnology and Biomolecular Sciences, University of New South Wales, Kensington, Australia

## Abstract

Proteins of the Homeodomain-Interacting Protein Kinase (HIPK) family regulate an array of processes in mammalian systems, such as the DNA damage response, cellular proliferation and apoptosis. The nematode *Caenorhabditis elegans* has a single HIPK homologue called HPK-1. Previous studies have implicated HPK-1 in longevity control and suggested that this protein may be regulated in a stress-dependent manner. Here we set out to expand these observations by investigating the role of HPK-1 in longevity and in the response to heat and oxidative stress. We find that levels of HPK-1 are regulated by heat stress, and that HPK-1 contributes to survival following heat or oxidative stress. Additionally, we show that HPK-1 is required for normal longevity, with loss of HPK-1 function leading to a faster decline of physiological processes that reflect premature ageing. Through microarray analysis, we have found that HPK-1-regulated genes include those encoding proteins that serve important functions in stress responses such as Phase I and Phase II detoxification enzymes. Consistent with a role in longevity assurance, HPK-1 also regulates the expression of age-regulated genes. Lastly, we show that HPK-1 functions in the same pathway as DAF-16 to regulate longevity and reveal a new role for HPK-1 in development.

Homeodomain interacting protein kinases (HIPKs) are nuclear serine/threonine kinases that phosphorylate a variety of targets including numerous transcription factors^1^,^2^. The functions of the mammalian members of this protein family (HIPK1-4) include roles in development and in regulation of cellular proliferation^3^,^4^. These proteins are also involved in modulating cellular responses to various stress stimuli including DNA damage^5^,^6^ and hypoxia^7^.

The genome of the nematode *Caenorhabditis elegans* encodes a single member of the HIPK protein family, called HPK-1^8^. We recently described a role for this protein in the control of proliferation of germ cells in *C. elegans* hermaphrodites^9^. In that report, we generated a transgenic *C. elegans* strain carrying a fosmid construct in which the *hpk-1* locus was recombineered to include a C-terminal mCherry tag in the encoded HPK-1 protein. Using this strain, we found that HPK-1 is broadly expressed in somatic cells. We detected only low levels of this fluorescently tagged HPK-1 under standard culture conditions, while increased fluorescence was observed after heat-shock treatment, suggesting that HPK-1 levels are regulated by heat stress^9^. Given the characterized roles of HIPK in several stress response pathways, this observation prompted us to consider whether HPK-1 contributes to the heat-shock response.

The heat-shock response involves activation of heat-shock factor -1 (HSF-1). This transcription factor drives the expression of numerous target genes including those that encode the small heat-shock proteins (HSPs)^10^. The HSPs are molecular chaperones that enhance protein folding and prevent aggregation of damaged proteins (reviewed in^11^). The induction of HSPs in response to heat-shock diminishes with age^12^,^13^ and thermotolerance is reduced in aged animals^13^,^14^, implicating the heat-shock response system in the ageing process. Consistent with this, in addition to causing sensitivity to heat stress, loss of function of HSF-1 shortens *C. elegans* lifespan and hastens tissue ageing^15^, while overexpression of HSF-1 extends lifespan^10^.

Beyond the heat-shock response, a general correlation between resistance to a variety of stressors and longevity has been widely noted (reviewed in^16^). For example, reduction of function of *mdt-15*, which encodes a component of the transcription regulatory Mediator complex, results in shortened lifespan^17^ and sensitivity to oxidative stress^18^. Conversely, long-lived *daf-2* mutants^19^ show resistance to heat^20^ and oxidative stress^21^. *daf-2* encodes the insulin/insulin-like growth factor receptor, which regulates longevity and stress responses through control of the transcription factors DAF-16^22^ and SKN-1^23^. Interestingly, *hpk-1* was identified in a genome-wide screen as one of 41 genes that when knocked down by RNA interference (RNAi) reduced the extended lifespan of *daf-2* mutants, but did not affect the lifespan of *daf-2; daf-16* mutant animals^24^, suggesting HPK-1 as a potential ageing regulator.

Here we confirm that HPK-1 levels are increased following heat-shock and show that loss of function of HPK-1 renders worms hypersensitive to heat and oxidative stress. Loss of HPK-1 function also shortens lifespan and hastens physiological decline during aging. In addition, we present microarray analyses comparing transcripts from wild type and *hpk-1* mutant animals. Through these analyses we find that HPK-1 regulates the expression both of genes that play important roles in stress responses and of genes that are age-regulated.

## Results

### HPK-1 protein levels increase following heat-shock

**Figure 1:**
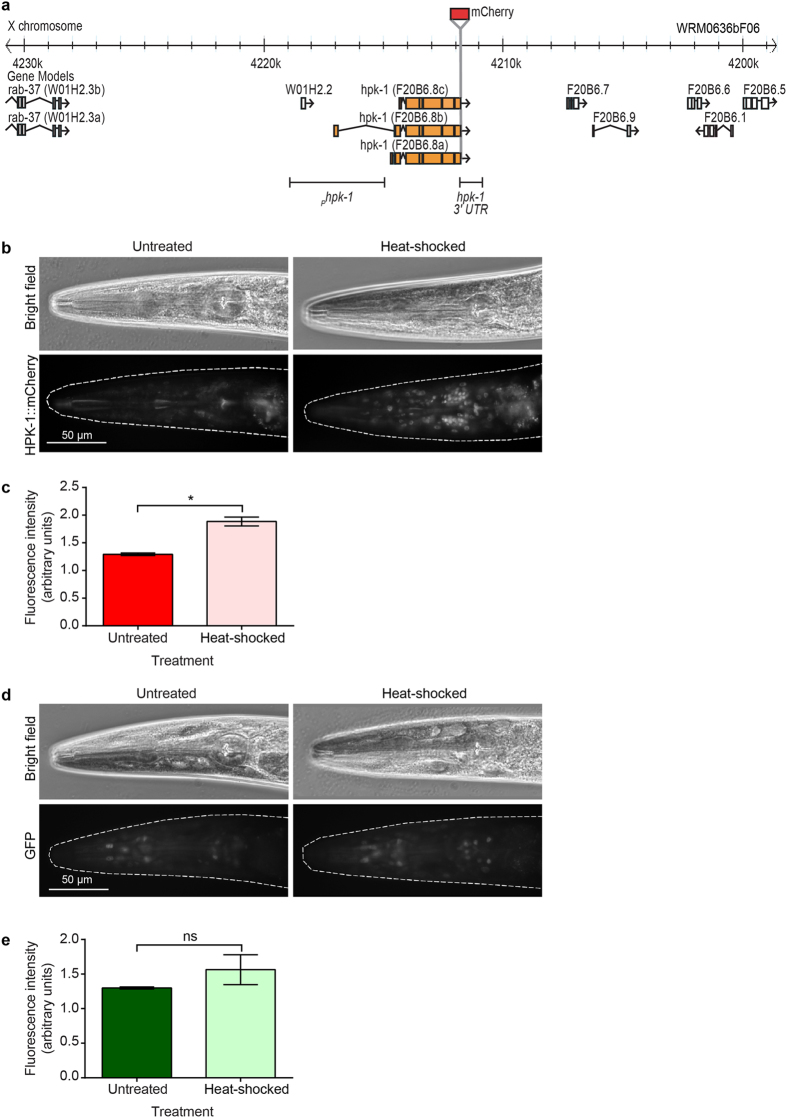
HPK-1 levels increase following heat-shock. (**a**) mCherry optimised for *C. elegans* expression was inserted in-frame at the C-terminus of the *hpk-1* gene within fosmid WRM0636bF06. (**b**) Representative bright field and fluorescence images of the head region of untreated and heat-shocked transgenic *ausIs6*[*hpk-1::mCherry*] adult animals. (**c**) Fluorescence intensity of HPK-1::mCherry in the head area quantified using ImageJ, shown as fold change normalised to non-transgenic wild type animals. Graphs show mean ± SEM of three independent experiments. n = 10 per strain per replicate. Unpaired t-test, two tailed; *p < 0.05. (**d**) Representative bright field and fluorescence images of the head region of untreated and heat-shocked transgenic *ausIs27* [_*P*_*hpk-1::GFP::H2B::hpk-1 3*′ *UTR*] adult animals. Genomic DNA regions used for _*P*_*hpk-1* and *hpk-1 3*′ *UTR* are indicated in (**a)**. (**e**) Fluorescence intensity of GFP in the head area quantified using ImageJ, shown as fold change normalised to non-transgenic wild type animals. Graphs show mean ± SEM of three independent experiments. n = 10 per strain per replicate. Unpaired t-test, two tailed; ns = not significant.

We previously showed that expression of a fosmid transgene containing HPK-1 tagged with mCherry (*ausIs6*[*hpk-1::mCherry*]) is low in a variety of somatic cells under normal culture conditions^9^. As the *ausIs6* transgene contains the entire *hpk-1* genomic locus, including 13.5 kb upstream and 12 kb downstream of the *hpk-1* coding sequence (WRM0636bF06), this will hereafter be referred to as *Is*[*hpk-1*(+)] (Fig. 1a). Interestingly, we noted a qualitative increase in mCherry fluorescence when animals carrying this transgene are heat-shocked^9^, suggesting that HPK-1 expression or stability may be altered by heat stress. To investigate this further, we quantified mCherry fluorescence in animals carrying the *ausIs6* transgene under normal culture conditions and following an acute heat-shock (37 °C for 1 hour). Measuring fluorescence intensity revealed a 1.5 fold increase in heat-shocked animals compared with untreated animals (Fig. 1b,c), suggesting that HPK-1 protein levels are increased after heat-shock. The observed increase in HPK-1::mCherry fluorescence might be due to higher levels of transcription from the *hpk-1* promoter, or might instead reflect a change in translation, stability or localisation of the tagged HPK-1 protein.

To test whether transcription from the *hpk-1* promoter is regulated by heat-shock, we generated a strain carrying a reporter transgene from which GFP fused to the histone protein H2B is expressed under the control of the putative *hpk-1* promoter (*ausIs27*[_*P*_*hpk-1::GFP::H2B::hpk-1 3*′ *UTR*]). With the goal of incorporating all likely transcriptional cis-regulatory elements, we used for this purpose a genomic fragment encompassing 1419 bp upstream of the annotated 5′ UTR of the *hpk-1* coding sequence together with the first exon and 2210 bp of the first intron of the longest predicted *hpk-1* isoform (F20B6.8b) (Fig. 1a). GFP fluorescence in animals carrying the *ausIs27* transgene was quantified under normal culture conditions and following an acute heat-shock (37 °C for 1 hour). No significant difference in fluorescence between these two conditions was observed (Fig. 1d,e), indicating that transcription from the *hpk-1* promoter is not altered by heat-shock treatment. While it remains possible that certain sequences required for native transcriptional regulation of *hpk-1* lie outside the 4016 bp genomic region that we employed in these experiments, our observations suggest that heat-shock affects HPK-1 levels through a post-transcriptional mechanism.

### *hpk-1*(−) animals display enhanced sensitivity to heat stress and oxidative stress

**Figure 2:**
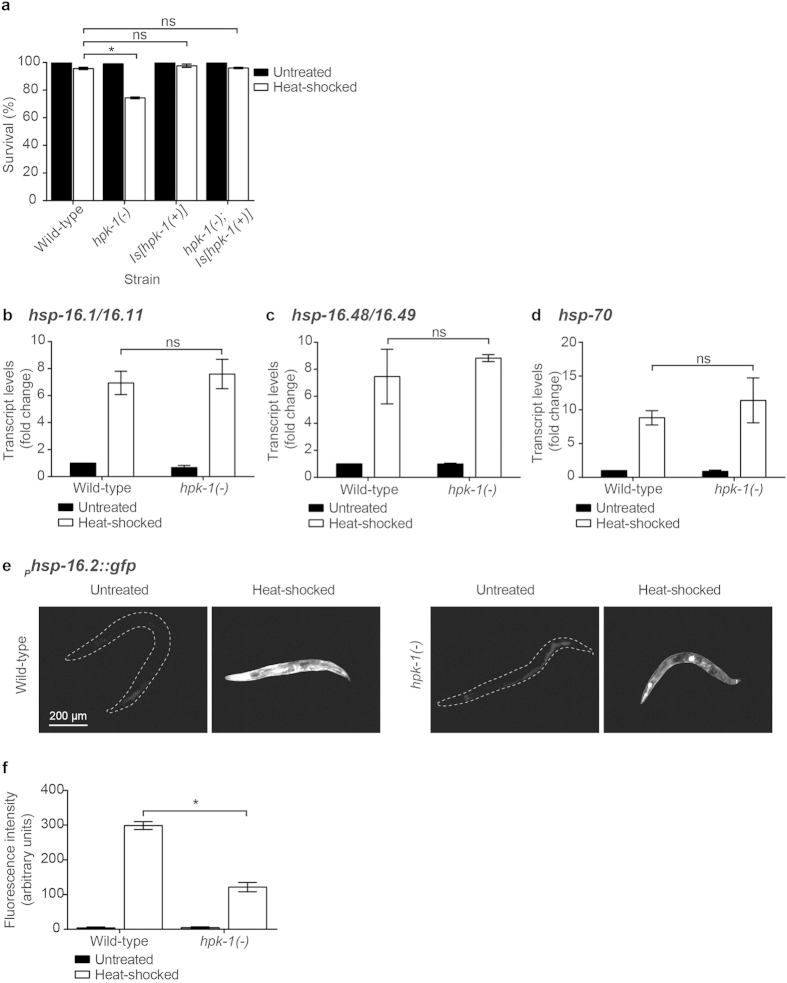
Loss of HPK-1 function confers sensitivity to heat stress. (**a**) Survival of wild type, *hpk-1*(−)*, Is*[*hpk-1*(+)] and *hpk-1*(−)*; Is*[*hpk-1*(+)] animals 24 hours after heat-shock treatment (37.5 °C for 2 hours). Graph shows mean ± SEM of three independent experiments for heat-shocked data, n = 57–220 per experiment. Two-way ANOVA, Sidak’s multiple comparisons test, *p < 0.05; ns = not significant. (**b–d**) The levels of heat-shock protein (hsp) transcripts were quantified immediately following heat-shock treatment (37 °C for 1.5 hours) in wild type and *hpk-1*(−) animals and expressed as a fold change relative to the untreated wild type cohort. Levels of the following transcripts were quantified: (**b)** *hsp-16.1/16.11*, (**c)** *hsp-16.48/16.49* and (**d)** *hsp-70*. Graphs show mean ± SEM of two independent experiments. Two-way ANOVA, Sidak’s multiple comparisons test; ns = not significant. (**e)** Representative micrographs showing animals with the median level of _*P*_*hsp-16.2::gfp* expression in untreated and heat-shocked wild type (left) and *hpk-1*(−) animals (right) 24 hours post heat-shock treatment (37 °C for 1.5 hours). (**f)** Fluorescence intensity of GFP in the whole animal 24 hours post heat-shock treatment (37 °C for 1.5 hours), quantified using ImageJ. Graph shows mean ± SEM of three independent experiments. n = 10 per strain per replicate. Two-way ANOVA, Sidak’s multiple comparisons test, *p < 0.05.

We next investigated whether HPK-1 is required for animals to withstand heat stress. Strain EK273 *hpk-1*(*pk1393*) was used for these investigations. The *pk1393* allele is a 1457 bp deletion that removes most of the kinase domain and is predicted to be a null allele^8^. Animals carrying the *pk1393* allele will henceforth be referred to as *hpk-1*(−). Wild type and *hpk-1*(−) animals were exposed to an acute heat-shock (37.5 °C for 2 hours) and scored for survival 24 hours after the treatment. While survival of wild type animals was only mildly affected by this heat-shock treatment (96 ± 1%; mean ± SEM), *hpk-1*(−) animals showed significantly increased sensitivity, with 74 ± 1% survival (Fig. 2a). To confirm that this reduced survival is attributable to the loss of *hpk-1* function, we assessed whether wild type survival could be restored in *hpk-1*(−) animals through the transgenic introduction of wild type copies of *hpk-1*. The survival of *hpk-1*(−) animals carrying the *Is*[*hpk-1*(+)] transgene (96 ± 1%) was not significantly different from that of wild type animals (Fig. 2a), indicating rescue of the *hpk-1*(−) phenotype by the *Is*[*hpk-1*(+)] transgene and confirming a role for HPK-1 in survival following heat stress.

We hypothesised that the canonical heat-shock response might be compromised in *hpk-1*(−) animals. To test this, we used real-time RT-PCR to measure the levels of several transcripts that encode HSPs, including small HSPs *hsp-16.1/16.11, hsp-16.48/16.49* and *hsp-70*, which have been reported to be induced in wild type animals following heat-shock^10^. Consistent with published observations, increased levels of these transcripts were detected in wild type animals after heat-shock treatment (37 °C for 1.5 hours) compared with untreated controls (Fig. 2b–d). Interestingly, these transcripts were also increased following heat-shock treatment in *hpk-1*(−) animals, to the same level observed in wild type animals. HPK-1 is therefore not essential for the heat-shock regulated induction of these genes that encode HSPs.

To investigate further whether HPK-1 influences the induction of HSPs, we utilised a _*P*_*hsp-16.2::gfp* transcriptional reporter (*ncIs17* transgene). Expression from this reporter is undetectable under normal maintenance conditions but is reported to be strongly induced in the pharynx and intestine after heat stress^25^. Consistent with this, we observed a marked increase in GFP fluorescence from this reporter in wild type animals 24 hours after heat-shock treatment (Fig. 2e,f). In *hpk-1*(−) animals carrying this reporter, GFP fluorescence also increased after heat-shock treatment relative to untreated control animals. However, the GFP fluorescence in heat-shock treated *hpk-1*(−) animals was significantly lower than that observed in heat-shock treated wild type animals (Fig. 2e,f). Together with our real-time RT-PCR data, these observations suggest that HPK-1 is not an essential component of the machinery that induces expression of the HSPs in response to heat stress. The reduced induction of expression from the *hsp-16.2* promoter in *hpk-1*(−) animals raises the possibility that HPK-1 may instead be a modulator of the scale of the heat stress response.

**Figure 3:**
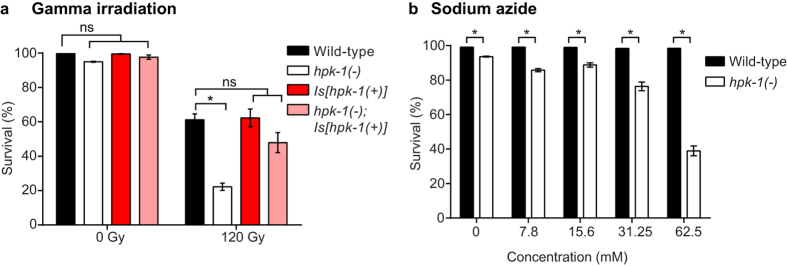
Loss of HPK-1 function confers sensitivity to oxidative stress. (**a**) Embryonic survival was quantified in the progeny of wild type, *hpk-1*(−)*, Is*[*hpk-1*(+)] and *hpk-1*(−)*; Is*[*hpk-1*(+)] animals treated with gamma irradiation. Graph shows mean ± SEM of three independent experiments. n = 40–50 per experiment. Two-way ANOVA, Sidak’s multiple comparisons test. (**b**) Wild type and *hpk-1*(−) animals were scored for survival after exposure to increasing concentrations of sodium azide. Graph shows mean ± SEM of three independent experiments. n = 119–210 per experiment. Two-way ANOVA, Sidak’s multiple comparisons test. For all panels *p < 0.05, ns = not significant.

While we have presented here the first evidence of a role for a metazoan member of the HIPK protein family in the response to heat stress, the mammalian HIPKs have previously been implicated in the response to other stress stimuli including DNA damage^5^,^6^. We therefore tested whether HPK-1 contributes to the DNA damage response in *C. elegans*. After DNA damage, cells in the *C. elegans* germline utilise different mechanisms to either repair the damage or eliminate the damaged cell^26^,^27^. In the distal part of the germline where cells are undergoing mitotic divisions, damaged cells arrest to allow for repair, while damaged cells at the pachytene stage of meiosis can undergo apoptosis^28^. If a germ cell with DNA damage is not eliminated through apoptosis or the damage is not repaired, these cells give rise to inviable embryos. We therefore analysed cell cycle arrest, germline apoptosis and embryonic survival following gamma irradiation to examine whether HPK-1 is required for the response to DNA damage. Like wild type, *hpk-1*(−) animals displayed cell cycle arrest and apoptosis in the mitotic and pachytene regions of the germline, respectively, after exposure to 120 Gy dose of gamma irradiation (Figure S1). These observations suggest that, unlike mammalian HIPKs, *C. elegans* HPK-1 is dispensable for the DNA damage response. However, we detected a marked reduction in the survival of the progeny of *hpk-1*(−) animals after gamma irradiation at the young adult stage. In the absence of gamma irradiation, the embryonic survival of wild type and *hpk-1*(−) animals was 100 ± 0% and 95 ± 0%, respectively (Fig. 3a). Following treatment with gamma irradiation, embryonic survival reduced to 61 ± 3% in wild type animals and was significantly lower, at 22 ± 2%, in *hpk-1*(−) animals, indicating that loss of *hpk-1* function renders animals hypersensitive to gamma irradiation-induced embryonic lethality (Fig. 3a). This phenotype of the *hpk-1*(−) mutant animals was rescued with the *Is*[*hpk*(+)] transgene, with embryonic survival in *hpk-1*(−)*; Is*[*hpk-1*(+)] animals observed to be 48 ± 6%, which was not significantly different from wild type embryonic survival (Fig. 3a). Thus HPK-1 plays a role in embryonic survival following gamma irradiation. Since HPK-1 does not appear to function in the DNA damage response, this finding may instead reflect a requirement for HPK-1 in responding more generally to the oxidative stress that is caused by gamma irradiation^29^.

To further assess a potential role for HPK-1 in the oxidative stress response, we utilised sodium azide, a cytochrome oxidase inhibitor, to induce oxidative stress via inhibition of the mitochondrial electron transport chain^30^. Survival after exposure to increasing concentrations of sodium azide was scored in wild type animals and *hpk-1*(−) animals. In the conditions used for these experiments (7.8–62.5 mM sodium azide, 90 minute exposure), survival of wild type animals was not significantly affected by sodium azide (Fig. 3b). *hpk-1*(−) animals showed reduced survival compared with wild type at all tested concentrations. For example, while 98 ± 0% of wild type animals survived exposure to 62.5 mM sodium azide, only 39 ± 3% of *hpk-1*(−) animals survived (Fig. 3b). This supports the conclusion that HPK-1 plays a role in the response to oxidative stress.

### HPK-1 regulates longevity

**Figure 4:**
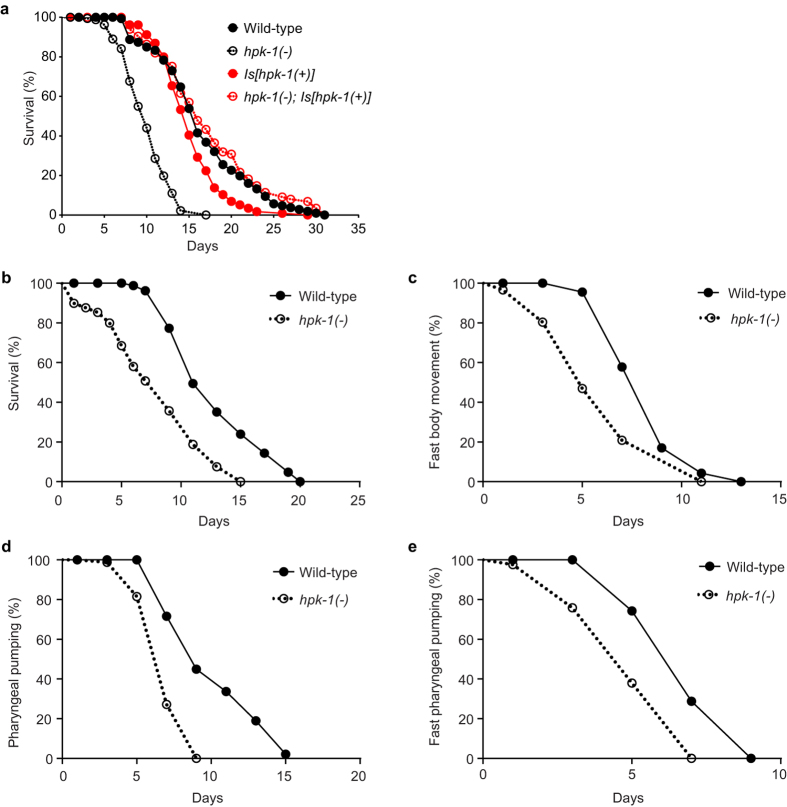
Loss of HPK-1 function results in lifespan reduction and accelerated decline of physiological processes. (**a**) Survival curves of wild type, *hpk-1*(−)*, Is*[*hpk-1*(+)] and *hpk-1*(−)*; Is*[*hpk-1*(+)] animals at 20 °C. n = 170 per strain at day 0. Log-rank test, p < 0.0001 for wild type vs *hpk-1*(−), p < 0.01 for wild type vs *Is*[*hpk-1*(+)], n.s. for wild type vs *hpk-1*(−)*; Is*[*hpk-1*(+)]. (**b–e**) Synchronous populations of wild type and *hpk-1*(−) animals maintained at 22.5 °C were monitored for (**b**) survival, (**c**) fast body movement, (**d**) pharyngeal pumping, and (**e**) fast pharyngeal pumping. For (**b–e**), n > 90 per strain at day 0. Log-rank test, p < 0.0001 for all (**b–e**).

It has been well documented that lifespan and stress sensitivity often correlate in *C. elegans*, with mutants that are stress sensitive commonly displaying shortened lifespan and vice versa (reviewed in^16^). The finding that *hpk-1*(−) animals are sensitive to heat and oxidative stress therefore prompted us to consider whether longevity is also influenced by loss of HPK-1 function. As mentioned previously, HPK-1 had been implicated in lifespan regulation through a genome-wide RNAi screen in which *hpk-1*(*RNAi*) reduced the lifespan of long-lived *daf-2* mutants and of wild type animals^24^. To corroborate this finding, we examined the lifespan of *hpk-1*(−) mutants. In two independent lifespan assays, *hpk-1*(−) animals displayed significantly reduced lifespan compared with wild type animals. Median lifespan for *hpk-1*(−) animals was 9 or 10 days while that of wild type animals was 15 or 17 days (p < 0.001, Log-rank test) (Figure S2a,b). To confirm that this reduction in lifespan was attributable to loss of *hpk-1* function, we compared the lifespan of wild type and *hpk-1*(−) animals with the lifespan of animals carrying the *Is*[*hpk-1*(+)] transgene. Compared with the median lifespan of 10 days for *hpk-1*(−) animals, wild type animals and *hpk-1*(−)*; Is*[*hpk-1*(+)] animals displayed median lifespans of 16 and 15 days, respectively (Fig. 4a and S2c). This restoration of wild type lifespan in *hpk-1*(−) mutants by the *Is*[*hpk-1*(+)] transgene confirms a role for HPK-1 in lifespan regulation.

To examine whether the observed lifespan reduction in *hpk-1*(−) animals is indicative of precocious ageing, several age-related phenotypes were scored in a longitudinal study. Body movement and pharyngeal pumping are two physiological processes in *C. elegans* in which a decline has previously been shown to positively correlate with ageing^31^. These phenotypes were monitored over the lifetime of 65 individual wild type and *hpk-1*(−) animals. Consistent with previous assays (Fig. 4a and S2), the lifespan of *hpk-1*(−) animals in this assay was also reduced compared with that of wild type (mean lifespan ± SD, 8.4 ± 3.8 days vs 12.9 ± 3.8 days, p < 0.0001, Log-rank test) (Fig. 4b). Additionally, body movement and pharyngeal pumping declined more rapidly in *hpk-1*(−) animals than in wild type animals as evidenced by significantly reduced fast body movement span (4.5 ± 1.8 days vs 8.1 ± 1.6 days), fast pharyngeal pumping span (4 ± 1.3 days vs 6.5 ± 1.3 days) and pharyngeal pumping span (6.5 ± 1.5 days vs 9.7 ± 2.9 days) (p < 0.0001 for all three parameters, Log-rank test) (Fig. 4c–e). The more rapid decline of these physiological parameters suggests that the lifespan reduction observed in *hpk-1*(−) animals is indicative of accelerated ageing.

### HPK-1-regulated genes include stress-related genes and are enriched for intestinal expression

The majority of proteins that are known to be phosphorylated, and hence regulated, by members of the HIPK family are involved in gene transcription (reviewed in^2^). Reasoning that nematode HPK-1 is likely to similarly regulate transcription, we predicted that loss of HPK-1 function would result in significant changes in gene expression. Therefore, to gain further insight into how HPK-1 may be contributing to heat and oxidative stress responses and lifespan regulation, we compared the transcriptional profiles of wild type animals and *hpk-1*(−) animals at the young adult stage using Affymetrix GeneChip microarrays. The expression data were analysed using Chipster® software to identify genes that are differentially expressed between the two worm strains, indicating HPK-1-dependent regulation. In total 123 genes showed decreased expression and 166 genes showed increased expression, by 1.5 fold or more, in *hpk-1*(−) animals compared with wild type populations (p < 0.05; Table S1 and S2). The DAVID gene ontology (GO) annotation tool was then used to identify biological pathways or protein domains that are enriched in these differentially expressed gene sets to clarify the physiological processes that are regulated by HPK-1.

Among the genes that show decreased expression in *hpk-1*(−) animals, 48 were classified using the biological process ontology. The most highly-represented biological process is oxidation reduction with 14 genes (11.6% of analysed genes) involved in this process, including two short chain dehydrogenases *dhs-14* and *dhs-23* (Table 1). Interestingly two other enriched categories identified in this analysis include response to abiotic stimulus (5 genes or 4.1% of total analysed genes) and response to temperature stimulus (3 genes or 2.5% of analysed genes). Since temperature is an abiotic stimulus, all 3 genes identified in the response to temperature stimulus category (*mtl-1, hsp-12.3* and *fat-7*) are also in the response to abiotic stimulus category, which further includes *gpdh-1* and the glutathione S transferase *gst-10*.

Further classification of 90 of the genes that show decreased expression in *hpk-1*(−) by INTERPRO domains revealed enrichment of UDP-glucuronosyl/UDP-glucosyltransferases (UGTs) and Cytochrome P450s, in addition to several uncharacterised protein families or domains of unknown function (Table 2). UGTs and cytochrome P450s are functionally relevant to stress responses, with both being involved in detoxification in response to endo- and xenobiotic stimuli (reviewed in^32^).

With regard to the genes that showed increased expression in *hpk-1*(−) animals, 50 were classified using the biological process ontology. The most highly-represented biological processes are growth and body morphogenesis with a total of 18 genes involved in these two processes (Table 1). Other significantly enriched biological processes in this gene set include neuropeptide signalling, protein dephosphorylation and cuticle development (Table 1). Classification of 103 of the genes that show increased expression in *hpk-1*(−) animals by INTERPRO domains identified enrichment for only one domain, TB2/DP1 and HVA22 related protein. Out of 5 *C. elegans* genes in this category, 2 were found to have increased expression in *hpk-1*(−) animals (% of total set = 1.21%, p = 3.68 ×10^−2^).

We reasoned that identifying the tissues in which HPK-1-regulated genes are expressed might provide further information on the potential mechanisms through which HPK-1 regulates stress responses and ageing. We therefore compared the 289 genes found to be regulated in a HPK-1-dependent manner in our study with published datasets documenting transcripts enriched in the intestine, muscle, nervous system and germline^33^,^34^,^35^,^36^. No significant overlap was found between HPK-1-regulated genes and those enriched in muscle, germline or neurons (Table 3). In contrast, 30 intestine-enriched transcripts are among the HPK-1-regulated genes, which is a significant overrepresentation (Table 3). These intestine-enriched transcripts include those that are both decreased (17) and increased (13) in *hpk-1*(−) animals (Table S3 and S4). This finding is of particular interest because the intestine is a major site of response to environmental stress (reviewed in^37^).

### The expression of age-regulated genes such as *sod-3* and *ugt-9* is regulated by HPK-1

**Figure 5:**
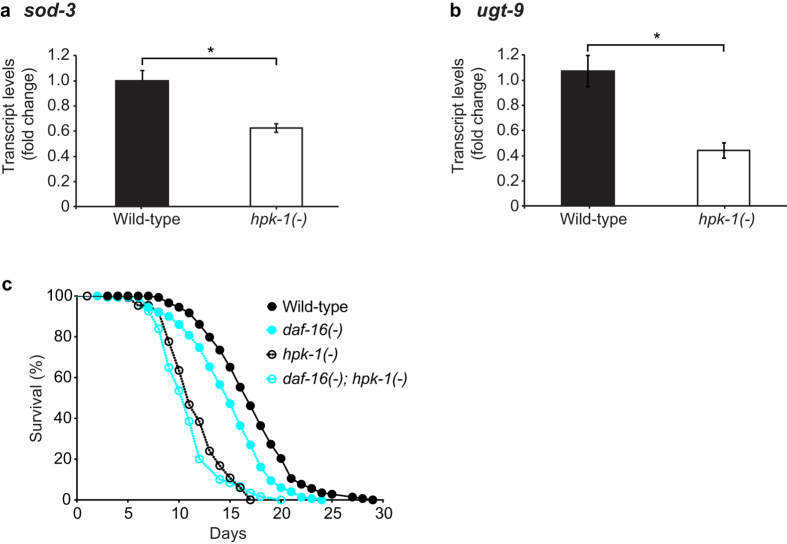
HPK-1 regulates expression of genes that are regulated by the IIS pathway. (**a,b**) Transcript levels were quantified in wild type and *hpk-1*(−) animals and expressed as a fold change relative to the wild type cohort. Levels of the following transcripts were quantified: (**a**) *sod-3* and (**b**) *ugt-9*. Graphs show mean ± SEM of three independent experiments. Unpaired t-test, p-value is indicated by *p < 0.05. (**c)** Survival curve of wild type, *daf-16*(−), *hpk-1*(−) and *daf-16*(−)*; hpk-1*(−) animals at 20 °C. n = 200 per strain at day 0. Log-rank test, p < 0.0001 for wild type vs daf-16(−), wild type vs *hpk-1*(−) and wild type vs *daf-16*(−)*; hpk-1*(−), n.s. for *hpk-1*(−) vs *daf-16*(−)*; hpk-1*(−). daf-16(−) denotes the *daf-16*(mu86) allele.

Given our finding that loss of *hpk-1* function results in shortened lifespan, we additionally investigated the overlap between HPK-1 regulated genes and known age-regulated genes^38^. Genes that show both increased and decreased expression with age had been identified using transcriptional profiling throughout an ageing time course^38^. We found a statistically significant overlap between age-regulated genes and genes that are regulated in a HPK-1-dependent manner. Specifically, 39 age-regulated genes are also regulated by HPK-1, including 22 genes that show decreased expression in *hpk-1*(−) animals and 17 genes that show increased expression in *hpk-1*(−) animals (Table 3, **S5** and **S6**). Budovskaya *et al.* (2008) found that a large number of genes that change expression with age are regulated by *elt-3*, a GATA transcription factor. Using GFP reporters, *elt-3* and two of its targets, the putative UDP-glucuronosyltransferase *ugt-9* and the Fe/Mn superoxide dismutase *sod-3*, were shown to decrease in expression with age^38^. Interestingly, both *elt-3* and *ugt-9* showed significantly decreased expression in *hpk-1*(−) animals. *sod-3* was also reduced in *hpk-1*(−) animals, but with a p-value marginally higher than the threshold set for statistical significance (p = 0.078). Given that the *sod-3* expression change observed through microarray analysis was not statistically robust, we used real-time RT-PCR to re-examine the levels of both *sod-3* and *ugt-9* in RNA extracted from independently-cultured populations of worms and found that both transcripts were indeed reduced in *hpk-1*(−) animals compared with wild type (Fig. 5a,b).

### HPK-1 regulates genes that are also regulated by the IIS pathway

*sod-3* is a well-studied target of the insulin/IGF-1 signalling (IIS) pathway^21^ and multiple members of the UGT family, to which *ugt-9* belongs, are similarly regulated by IIS^39^. In light of this and the previous report that HPK-1 is required for *daf-2*(−)-dependent lifespan extension^24^, we decided to examine further the relationship between HPK-1 and the IIS pathway. To this end, we first compared HPK-1 regulated genes with those that are regulated by the IIS pathway. From the numerous studies that have catalogued genes that are regulated by the IIS pathway we selected one dataset for this comparison. In this dataset are IIS target genes that were identified through microarray analysis as transcripts that are increased in long-lived *daf-2*(*RNAi*) animals and decreased in *daf-16*(*RNAi*)*;daf-2*(*RNAi*) animals that have normal lifespan^40^. Among 259 IIS target genes, 8 showed decreased expression and 7 showed increased expression in *hpk-1*(−) animals (Table 3, S7 and S8). These include the fatty acid desaturase *fat-7*, the intestinal cysteine protease-related gene *cpr-1*^41^ and the MAPK phosphatase *vhp-1*^42^, which are also age-regulated genes^38^.

We next examined the effect of a *daf-16*(*mu86*)*; hpk-1*(−) double mutation on lifespan. In two independent lifespan assays, we observed that *daf-16*(*mu86*)*; hpk-1*(−) animals had a median lifespan of 11 days, which was not significantly different from that of *hpk-1*(−) animals (11 or 10 days, respectively) (Fig. 5c and S5). This suggests that HPK-1 and DAF-16 act within the same pathway to regulate lifespan as no additive effects were observed. We also intended to examine whether, like RNAi knockdown of *hpk-1*, mutation of *hpk-1* would affect the lifespan extension afforded by *daf-2* mutation. We attempted to generate *daf-2*(*e1370*)*; hpk-1*(−) mutants but found that these animals were not viable. From parents that were homozygous for the *daf-2*(*e1370*) mutation and heterozygous for the *hpk-1*(−) mutation, 16% of progeny arrested as embryos when cultured at 20 °C (n = 1110). Arrested embryos from these assays were genotyped for the *hpk-1* allele and were found to be homozygous for the *hpk-1*(−) mutation. Embryonic arrest is rarely seen at this temperature in *daf-2*(*e1370*) single mutants (0% arrest, n = 182) or *hpk-1(*−) single mutants (1% arrest, n = 211). While the embryonic arrest of the *daf-2*(*e1370*)*; hpk-1*(−) double mutants precluded the analysis of the adult phenotype of longevity, the synthetic lethality of *daf-2*(*e1370*) and *hpk-1*(−) reveals a new role for *hpk-1* in development.

## Discussion

HPK-1 expression has been analysed in *C. elegans* using translational fluorescence reporters. The earliest report found that a HPK-1::GFP translational reporter is broadly expressed and localised to nuclear puncta during embryogenesis, with faint expression also observed in the adult head^8^. More recently, we observed low levels of a HPK-1::mCherry fusion protein under standard culture conditions throughout somatic tissues^9^. In mammalian systems, HIPK2 is reported to be a short-lived protein with a high turnover in unstressed cells^43^. Low levels of HIPK2 are maintained through interactions with the E3 ubiquitin ligase, seven in absentia homologue-1 (Siah-1), which targets HIPK2 for proteasomal degradation^43^. Our observation that HPK-1 levels are low in *C. elegans* may indicate that HPK-1 is negatively regulated in a similar manner to mammalian HIPKs under basal conditions.

HIPKs are regulated by a variety of stress stimuli. Under hypoxic conditions, mammalian HIPK2 is targeted for polyubiquitylation and proteasomal degradation^7^. Conversely, HIPK2 is stabilised following DNA damage through checkpoint kinase-mediated inhibition of ubiquitination^43^. Following oxidative stress, HIPK2 is de-SUMOylated and acetylated^44^. While the regulation of the mammalian HIPKs by hypoxia, DNA damage and oxidative stress has thus been well-documented, the regulation of a metazoan HIPK by heat stress has not previously been reported. Here we have shown that levels of the nematode HPK-1 protein increase after heat stress. This appears not to be due to increased transcription and may instead reflect stabilisation of the HPK-1 protein through post-translational modifications similar to those observed in mammalian HIPKs in response to other stress stimuli.

The heat stress-dependent regulation of HPK-1 protein levels suggests a role for HPK-1 in the response to heat stress. This notion is supported by our observation that loss of HPK-1 function reduces survival by ~25% following acute heat stress. However, since ~75% of *hpk-1*(−) animals can withstand a heat-shock treatment, HPK-1 does not appear to be essential for the heat-shock response, but rather, loss of *hpk-1* attenuates the response. One critical aspect of the protective response to heat-shock is the induction of expression of HSPs. While real-time RT-PCR analysis indicated no difference in the induction of several HSPs in *hpk-1*(−) mutants, the induction of a _*P*_*hsp-16.2::gfp* transcriptional reporter was reduced. Perhaps the heat stress conditions or the sensitivity of the real-time RT-PCR assay used were not sufficient to resolve a difference in the induction of *hsp-16.1/16.11, hsp-16.48/16.49* and *hsp-70*. Alternatively, HPK-1′s role in the heat shock response might be specific to the regulation of certain HSPs, including *hsp-16.2*. Further evidence that HPK-1 regulates the expression of small HSPs comes from our microarray analysis which found *hsp-12.3* (F38E11.1) to be reduced in *hpk-1*(−) mutants (Table S1).

How might HPK-1 regulate the response to heat-shock and expression of small hsps? HPK-1, along with other HIPKs, belongs to the dual specificity Yak1-related kinases (DYRK) family^8^,^45^. The ancestral yeast protein, Yak1, has, like its mammalian counterparts, been implicated in a range of stress responses. Importantly, Yak1 regulates the response to heat stress, with loss of function or overexpression of Yak1 rendering yeast sensitive or resistant, respectively, to heat-shock^46^. Furthermore, heat stress responsive genes have been identified that are regulated in a Yak1-dependent manner^46^,^47^. Interestingly, in response to glucose depletion, Yak1 phosphorylates the yeast homologue of HSF-1, Hsf1, enhancing DNA binding by this transcription factor^48^. Phosphorylation of mammalian HSF-1 has similarly been shown to influence DNA binding activity^49^. Following heat-shock in *C. elegans*, HSF-1 also undergoes post-translational modification^50^. These data from several systems raise the possibility that HPK-1 may contribute to the heat stress response via phosphorylation-mediated regulation of HSF-1.

In addition to a role for HPK-1 in the heat stress response, our data also indicate involvement of HPK-1 in the oxidative and xenobiotic stress responses. A role for HPK-1 in defence against oxidative damage is suggested by the elevated embryonic lethality of *hpk-1*(−) animals following gamma irradiation together with the increased sensitivity of *hpk-1*(−) animals to sodium azide treatment. Our microarray analysis revealed that the expression of both Phase I and Phase II detoxification enzymes is influenced by HPK-1. Phase I enzymes include the cytochrome P450s and the short chain dehydrogenases, while Phase II enzymes include the glutathione S transferases and UGTs. Representatives from each of these enzyme families showed decreased expression in *hpk-1*(−) mutants, suggesting a more general role for HPK-1 in defence against xenobiotic stress. Consistent with this is the intestinal enrichment of HPK-1-regulated genes, suggesting that HPK-1 acts at the main interface with the environment to modulate the response to xenobiotic stress. Other well-characterised stress response mediators such as SKN-1 and DAF-16 similarly act in the intestine to modulate stress responses^51^,^52^.

Aside from regulating stress responses, we have also explored the role of HPK-1 in longevity regulation. We found that loss of HPK-1 results in a shortened lifespan and accelerated physiological aging. These observations are in line with previous reports that *hpk-1*(*RNAi*) reduces the lifespan of wild type animals^24^. Through microarray analysis we have identified a significant overlap of genes that require HPK-1 for expression and genes that are age-regulated^38^, as well as between genes regulated by HPK-1 and genes induced by DAF-16^40^. These include some well-characterised DAF-16 targets such as *fat-7, hsp-12.3* and *mtl-1*^40^. Furthermore, we showed that loss of *daf-16* function in *hpk-1*(−) animals did not further shorten the lifespan of these animals, suggesting that HPK-1 and DAF-16 act in the same pathway for lifespan regulation. HPK-1 may therefore affect the activity of the IIS pathway to modulate longevity.

We additionally uncovered a developmental role for HPK-1 through the observation of synthetic embryonic lethality in *daf-2*(*e1370*); *hpk-1*(−) double mutants. The *e1370* allele is a reduction of function mutation that renders worms long-lived. However, *daf-2* null mutations result in embryonic lethality^53^ and the *e1370* allele also exhibits some embryonic lethality at 25 °C^54^. DAF-2 has therefore been proposed to serve distinct roles in different stages of life, promoting growth to adulthood and shortening the lifespan of adult animals^55^. Our observation that *daf-2*(*e1370*); *hpk-1*(−) mutants arrest during embryogenesis suggests that HPK-1 works in concert with DAF-2 to regulate a critical developmental event.

## Materials and Methods

### Strain Information

Strains were cultured on standard Nematode Growth Media (NGM) seeded with OP50 bacteria and grown at 25 °C unless otherwise specified. Strains used: wild type Bristol **N2**, **EK273** *hpk-1*(*pk1393*) X, **HRN250** *ausIs6* [*hpk-1::mCherry* (WRM0636bF06)*; unc-119 *+* coel::gfp*]^9^, **HRN257** *ausIs6; hpk-1*(*pk1393*), **ST66** *ncIs17* [_*P*_*hsp-16.2::eGFP*+ *pBluescript*], **HRN469** *ncIs17*; *hpk-1*(*pk1393*), **HRN442** *ausIs27* [_*P*_*hpk-1::GFP::H2B::hpk-1 3*′ *UTR *+* *_*P*_*myo-2::mCherry*], **CF1038** *daf-16*(*mu86*) I, **HRN252** *daf-16*(*mu86*)*; hpk-1*(*pk1393*), and **HRN153** *daf-2*(*e1370*) III.

### Generation of a line containing a *hpk-1* promoter-driven fluorescence reporter

A _*P*_*hpk-1::GFP::H2B::hpk-1 3*′ *UTR* expression construct was generated using the Gateway® Cloning System (Life Technologies). The putative promoter of the *hpk-1* gene (corresponding to the region between nucleotide 2966 of cosmid W01H2 and nucleotide 36852 of cosmid F20B6) and the *hpk-1 3*′ *UTR* (corresponding to nucleotides 33865-32810 of cosmid F20B6) were amplified from N2 genomic DNA and the amplicons were incorporated into the Gateway pDONRP4-P1R and pDONRP2R-P3 vectors, respectively, in BP reactions to generate pSB009 and pMW008. A LR reaction was then performed with pSB009, a middle entry clone (pCM1.35 (GFP-H2B in pDONR201; Addgene plasmid 17248)), pMW008, and the destination plasmid pBCN40-R4R3 (Addgene plasmid 34915)^56^. ~10 μg of the resulting plasmid pMW007 (_*P*_*hpk-1::GFP::H2B::hpk-1 3*′ *UTR *+* *_*P*_*myo-2::mcherry*) was introduced into young adult animals by biolistic transformation using the PDS-1000/He™ particle delivery system with a Hepta adaptor (BioRad, USA) according to the manufacturer’s instructions. A transgenic line was isolated using the dual antibiotic selection protocol^57^ and backcrossed to wild type (N2) six times to generate strain HRN442.

### Analysis of fluorescent reporter lines

Synchronous populations of worms were obtained by a timed egg-lay using gravid adults for 1 hour as described^58^. Animals were maintained at 20 °C until late into the fourth larval stage (L4) (66 hours post egg-lay), when animals to be heat-shocked were moved to 37 °C for 1 hour. Both untreated and treated animals were then moved to room temperature for 1 hour before being mounted for microscopy and imaged using a BX51 Microscope (Olympus). The sample was positioned using bright field microscopy to focus on the pharyngeal grinder. Micrographs were captured using AnalySIS software (Olympus) with fixed exposure times for fluorescence images. Images were analysed with ImageJ. Fluorescence was quantified in the region between the anterior tip of the worm and the posterior end of the terminal bulb of the pharynx in order to avoid interference from intestinal auto-fluorescence. The average *mCherry* or *GFP* fluorescence intensity in strains HRN250 and HRN442, respectively, was then expressed relative to background as measured in non-transgenic animals (N2) that had undergone the same treatment and image analysis. Imaging and fluorescence quantification was conducted blind to the genotype and treatment of the strains.

For _*P*_*hsp-16.2::gfp* image analysis, animals were maintained at 20 °C until young adulthood (24 hours after L4), when animals to be heat-shocked were moved to 37 °C for 1.5 hours. Animals were returned to 20 °C and imaged 24 hours later. Fluorescence intensity of the whole animal was measured using ImageJ.

### Heat stress survival assays

Synchronous populations of worms were obtained by allowing the eggs obtained from akaline hypochlorite treatment of gravid hermaphrodites^59^ to hatch in M9 buffer on a shaker overnight. The arrested first larval stage (L1) animals were then placed on OP50-seeded NGM plates and grown at 25 °C until young adulthood (24 hours after L4). Untreated populations were moved to 20 °C, while treated (heat-shocked) populations were placed at 37.5 °C for 2 hours and then allowed to recover at 20 °C for 24 hours before the number of live animals were scored. Animals were defined as alive if movement was observed after gentle prodding with an eyelash.

### Real-time reverse transcriptase (RT) PCR

Synchronous populations of worms were established as above. For real-time RT-PCR assays untreated animals were kept at 20 °C degrees, while treated (heat-shocked) animals were incubated at 37 °C for 1.5 hours. Animals were then frozen in liquid nitrogen before being crushed with a plastic pestle. The QIAshredder kit (Qiagen), RNeasy mini kit (Qiagen) and DNA-free™ DNase Treatment and Removal kit (Life Technologies) were used to extract RNA and remove DNA, according to the manufacturer’s instructions. cDNA was synthesised from approximately 3-10 μg of purified RNA, using the SuperScript™ III First-Strand cDNA synthesis kit (Life Technologies).Real-time PCR (cDNA, 2.5 μM primers, Fast SYBR® Green master mix) was performed using the 7500 Fast Real-Time PCR System (Life Technologies) with default settings. Transcript levels were calculated using the ΔΔCt method, with *act-1* as the normalisation control.

### Gamma irradiation survival assays

Synchronous populations of worms were established as above. Young adult populations were treated with 120 Gy gamma irradiation from a caesium 137 source. 24 hours after treatment, five gravid adults (per strain) were picked to lay eggs on each of ten fresh OP50-seeded NGM plates for 4 hours and then removed. Dead eggs and hatched larvae were scored on these plates 40–42 hours later.

### Sodium azide survival assays

Synchronous populations of worms were established by transferring the eggs obtained by alkaline hypochlorite treatment of gravid hermaphrodites onto OP50-seeded NGM plates. The worms were grown at 20 °C until early adulthood (48 hours after L4) then washed with M9 and transferred in M9 to siliconised tubes. The M9 was then replaced with M9 containing a range of concentrations of sodium azide (an electron transport chain inhibitor which chemically mimics hypoxia^60^, which had been prepared immediately prior to the assay). After incubating with gentle agitation for 80 minutes, the worms were allowed to sediment for 10 minutes and the supernatant removed before the pellet of worms was washed 3 times in M9. The washed worms were then transferred to NGM plates and scored as alive or dead after 24 hours recovery at 20 °C.

### Lifespan, body movement and pharyngeal pumping assays

To examine lifespan as well as physiological changes during ageing, synchronized animals were obtained by a timed egg-lay using gravid adults for 4 hours. When they reached the L4 stage, the hermaphrodites were picked onto OP50-seeded NGM plates and this was designated day 0. Animals were moved to fresh NGM plates every second day during the reproductive period to separate adults from their progeny. Assays were performed at 20 °C or 22.5 °C.

For lifespan assays, animals were scored daily and considered to be dead when they did not respond to gentle prodding with an eyelash. Animals that bagged (progeny hatched within the parent) or displayed vulval rupture were removed from the plates and censored from the analysis. Scoring and data analysis was performed blind to the genotype of the strains.

To examine physiological changes during ageing, the following parameters were scored every 2 days using a dissecting microscope: vital status, body movement and pharyngeal pumping. To determine vital status, animals were prodded gently with an eyelash and considered to be dead if they did not respond. Body movement and pharyngeal pumping were scored as previously described^31^.

### Microarray analysis

Animals were washed three times in M9 buffer and snap-frozen in TRI Reagent® (Sigma Chemical Company). Following two freeze thaw cycles, RNA was extracted through phase separation in TRI Reagent®. 100 ng of total RNA from duplicate wild type and *hpk-1*(−) mutant populations was amplified, labelled with biotin and fragmented using the GeneChip® 3′ IVT express kit according to the manufacturer’s instructions before hybridization to AffymetrixGeneChip® *C. elegans* Genome Arrays for 16 hours at 45 °C at 60 rpm in an Affymetrix hybridization oven. Arrays were washed according to the manufacturer’s instructions on an Affymetrix FS450 Fluidics Station and scanned on an Affymetrix Scanner GC3000 7G. Microarray data were then analysed with Chipster® software. The normalized intensities for each strain (calculated from the raw intensity values using the gcrma method) were averaged and fold changes in expression between strains were calculated. Changes with p < 0.05 were considered significant.

Genes that showed increased or decreased expression in *hpk-1*(−) were analysed for GO term enrichment using DAVID (the Database for Annotation, Visualization and Integrated Discovery) online tool. Tools available at http://www.nemates.org/ were used to compare gene lists and to determine the number of genes overlapping between two lists (observed) and the number of overlapping genes that would be expected if the genes were selected randomly (expected). The probability of finding the observed number of overlapping genes was also calculated. For these analyses, the number of unique transcripts covered by the AffymetrixGeneChip® *C. elegans* Genome Arrays was used as a reference, that is, 22150.

### Statistical analysis

The α-level is 0.05 for all analyses. Where appropriate, an unpaired, two-tailed Student’s t-test was used. For multiple comparisons, a two-way ANOVA with Sidak’s test was employed. Survival curves were analysed using a log-rank test to provide two-tailed p-values. The log-rank test, which gives equal weight to deaths at all time points, is commonly used for *C. elegans* lifespan data^31^. GraphPad Prism 6 (GraphPad Software Inc.) was used for all statistical analyses. Details of p-values are shown in Supplementary Table S9.

## Additional Information

**How to cite this article**: Berber, S. *et al.* Homeodomain-Interacting Protein Kinase (HPK-1) regulates stress responses and ageing in C. elegans. *Sci. Rep.* **6**, 19582; doi: 10.1038/srep19582 (2016).

## Supplementary Material

Supplementary Information

## Figures and Tables

**Table 1:** Occurrence of GO terms for biological processes in a set of genes that show decreased or increased expression in *hpk-1*(−) animals.

GO term	Count	% total	P-value
**Decreased Expression**			
Oxidation reduction	14	11.6	2.2 × 10^−7^
Lipid modification	5	4.1	4.3 × 10^−4^
Organic acid catabolic process	4	3.3	1.3 × 10^−3^
Response to abiotic stimulus	5	4.1	1.8 × 10^−3^
Phenol metabolic process	3	2.5	1.2 × 10^−2^
Cellular amino acid derivative metabolic process	3	2.5	1.3 × 10^−2^
Peptide metabolic process	3	2.5	1.9 × 10^−2^
Polyol catabolic process	2	1.7	2.4 × 10^−2^
Lipid glycosylation	3	2.5	2.8 × 10^−2^
Response to temperature stimulus	3	2.5	2.8 × 10^−2^
Fatty acid metabolic process	3	2.5	3.2 × 10^−2^
Cofactor metabolic process	4	3.3	4.3 × 10^−2^
**Increased Expression**			
Body morphogenesis	13	7.9	1.8 × 10^−4^
Collagen and cuticulin-based cuticle development	5	3.0	2.2 × 10^−3^
Molting cycle, collagen and cuticulin-based cuticle	6	3.6	1.9 × 10^−2^
Growth	15	9.1	3.6 × 10^−2^
Neuropeptide signaling pathway	3	1.8	4.2 × 10^−2^
Protein amino acid dephosphorylation	4	2.4	4.3 × 10^−2^
Regulation of multicellular organism growth	7	4.2	4.3 × 10^−2^

Genes that showed decreased or increased expression in *hpk-1*(−) animals compared with wild type animals were analysed for biological processes GO term enrichment using DAVID online analysis tool. Enriched GO terms are listed in the order of lowest to highest P-value. Out of 123 genes that decreased in *hpk-1*(−) animals, 48 were classified by this GO category. Out of 166 genes that increased in *hpk-1*(−) animals, 50 were classified by this GO category. ‘Count’ represents the number of genes in the dataset that are classified for the respective GO term. ‘% total’ is the percentage of the genes in the *hpk-1*(−) dataset that are represented by the GO term. P-value of 0.05 was chosen as a cut-off.

**Table 2:** Occurrence of INTERPRO domains in a set of genes that show decreased expression in *hpk-1*(−) animals.

INTERPRO domain	Count	%	P-value
Uncharacterised protein family UPF0376	5	4.1	5.3 × 10^−5^
UDP-glucuronosyl/UDP-glucosyltransferase	6	5.0	1.9 × 10^−4^
Protein of unknown function DUF19	5	4.1	2.9 × 10^−4^
Domain of unknown function, DUF-CC	3	2.5	3.1 × 10^−3^
Cytochrome P450	4	3.3	1.4 × 10^−2^
Glycoside hydrolase, subgroup, catalytic core	3	2.5	3.9 × 10^−2^

Genes that showed decreased expression in *hpk-1*(−) animals compared with wild type animals were analysed for INTERPRO domain enrichment using DAVID online analysis tool. Enriched INTERPRO domains are listed in the order of lowest to highest p-value. Out of 123 genes in this dataset 90 were involved in this annotation category. ‘Count’ represents the number of genes in the dataset that are classified for the respective domain. ‘% total’ is the percentage of the genes in the *hpk-1*(−) dataset that contain these protein domains. P-value of 0.05 was chosen as a cut-off.

**Table 3:** Comparison of *hpk-1*(−) microarray dataset with published datasets.

Published data sets	Down in *hpk-1*(−)123 genes	Up in *hpk-1*(*−*) 166 genes
Intestine enriched (624)Pauli *et al.*, 2006	Expected: 3Observed: 17p < 5.3 × 10^−8^	Expected: 5Observed: 13p < 7.6 × 10^−4^
Muscle enriched (230)Pauli *et al.*, 2006	Observed: 2ns	Observed: 2ns
Germline enriched (1135)Pauli *et al.*, 2006	Observed: 2ns	Observed: 13ns
Common to intestine, muscle and germline (510)Pauli *et al.*, 2006	Observed: 1ns	Observed: 1ns
Neuronal enriched (1625)Watson *et al.*, 2008	Observed: 9ns	Observed: 9ns
Ageing regulated (1254)Budovskaya *et al.*, 2008	Expected: 7Observed: 22p < 1.4 × 10^−6^	Expected: 9Observed: 17p < 1.3 × 10^−2^
Class 1 (DAF-16 induced) (259)Murphy *et al.*, 2003	Expected: 1Observed: 8p < 1.0 × 10^−4^	Expected: 2Observed: 7p < 3.0 × 10^−3^
Class 2 (DAF-16 repressed) (250)Murphy *et al.*, 2003	Observed: 0	Observed: 1

Online software (http://www.nemates.org/) was used to compare genes that show decreased or increased expression in *hpk-1*(*−*) animals with other published datasets. The number of genes in each dataset is indicated in brackets. Expected: the number of genes that would be expected to overlap between respective sets if genes were selected at random; Observed: the number of genes that were found to overlap between respective sets. ns = no significant difference.
