# Supplementary material for: Homeodomain-Interacting Protein Kinase (HPK-1) regulates stress responses and ageing in C. elegans
Source: Sci Rep. 2016 Jan 21;6:19582. doi: 10.1038/srep19582 (PMC4726358; doi:10.1038/srep19582)
Supplement: Supplementary Information [file srep19582-s1.pdf]

Supplementary Data

Title: Homeodomain-Interacting Protein Kinase (HPK-1) regulates stress responses and ageing in *C. elegans*

Authors: Slavica Berber[a], Mallory Wood[a], Estelle Llamosas, Priya Thaivalappil, Karen Lee, Mana Liao, Yee Lian Chew, Aaron Rhodes, Duygu Yucel, Merlin Crossley, Hannah R Nicholas[*]

* Corresponding author: Hannah Nicholas, School of Molecular Bioscience, University of Sydney, Sydney, NSW 2006, Australia. E-mail: hannah.nicholas@sydney.edu.au

[a] These authors contributed equally to this work.

Supplementary Figure S1. Loss of HPK-1 does not affect DNA damage response in the germline after gamma irradiation. (a) Representative images of DAPI-stained germlines in untreated animals (left) and 6 hours post gamma irradiation treatment (right). **(b)** Quantification of cells observed within 75 µm of the distal end of the germline. Graph shows mean ± SEM of two independent experiments. n = 10 per experiment. Two-way ANOVA, Sidak's multiple comparisons test *$p<0.05$. **(c)** Representative images of germline apoptotic regions stained with acridine orange in untreated (left) and 24 hours post gamma irradiation treatment (right). **(d)** Quantification of apoptotic corpses observed in the germline of untreated and gamma irradiated animals. Graph shows mean ± SEM of three independent experiments. n = 8-21 per experiment. Two-way ANOVA, Sidak's multiple comparisons test *$p<0.05$, ns = not significant.

Supplementary Figure S2. Loss of HPK-1 function results in a lifespan reduction. Replicate lifespan experiments showing that *hpk-1(-)* mutant animals have a significantly shortened lifespan compared with wild type controls. **(a,b)** Two independent experiments showing relative lifespans of wild type and *hpk-1(-)* animals maintained at 25 °C. n = 90 per strain at day 0. Log-rank test, $p<0.0001$ for wild type vs *hpk-1(-)* for both experiments. **(c)** Replicate of experiment shown in **Figure 4(a)**. n = 170 per strain at day 0. Log-rank test, $p<0.0001$ for wild type vs *hpk-1(-),* $p<0.01$ for wild type vs *Is[hpk-1(+)]* and wild type vs *hpk-1(-); Is[hpk-1(+)].*

Supplementary Figure S3. HPK-1 involvement in the IIS Pathway. Replicate of experiment shown in **Figure 5 (c)**. n = 170 per strain at day 0. Log-rank test, $p<0.0001$ for wild type vs *hpk-1(-)* and wild type vs *daf-16(-); hpk-1(-),* $p<0.01$ for wild type vs daf-16(-), n.s. for *hpk-1(-)* vs *daf-16(-); hpk-1(-).*

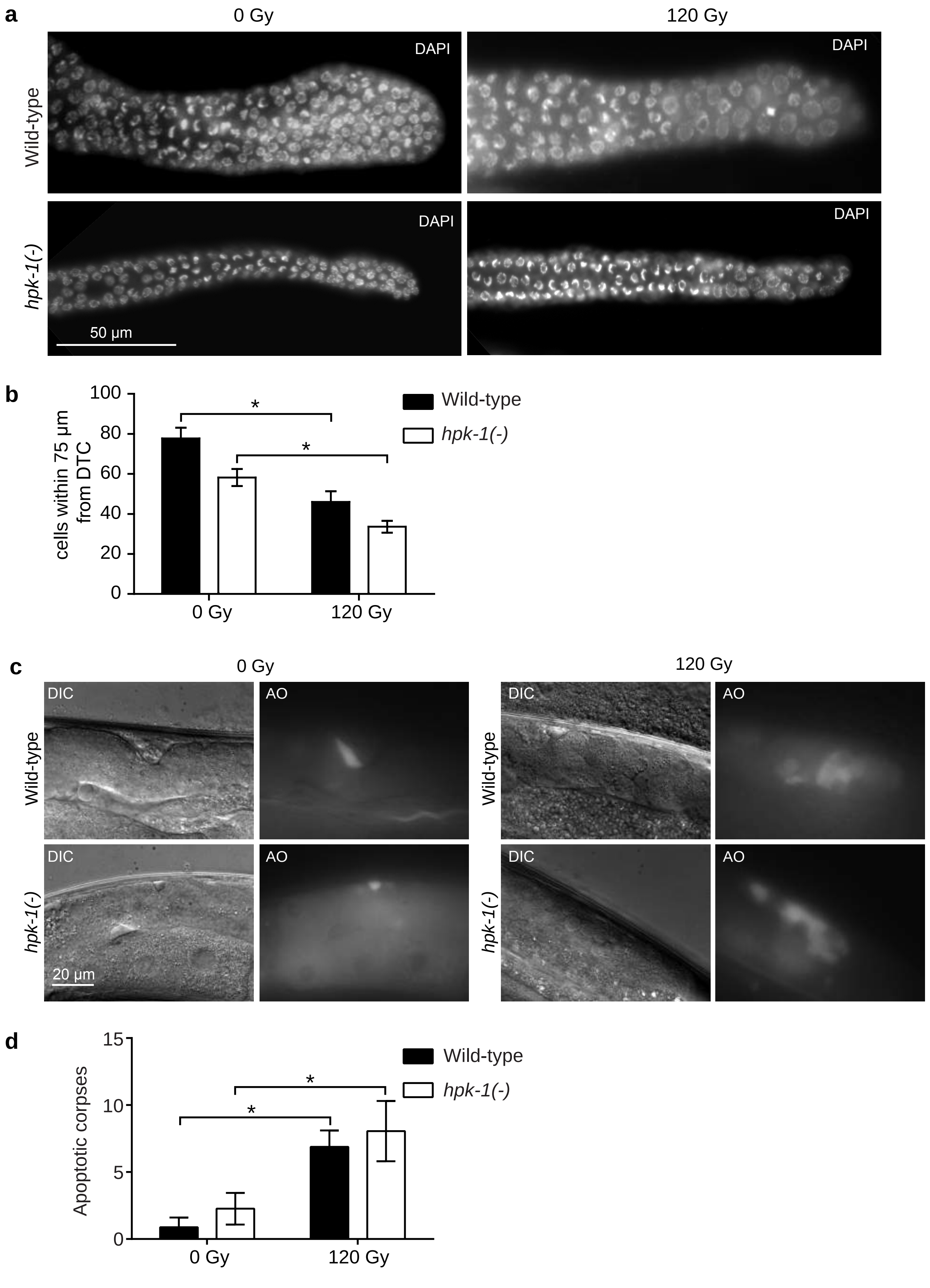
a
0 Gy
120 Gy
DAPI
Wild-type
hpk-1(-)
50 µm
b
cells within 75 µm from DTC
100
80
60
40
20
0
*
*
Wild-type
hpk-1(-)
0 Gy
120 Gy
c
0 Gy
120 Gy
DIC
AO
Wild-type
hpk-1(-)
20 µm
d
Apoptotic corpses
15
10
5
0
*
*
Wild-type
hpk-1(-)
0 Gy
120 Gy

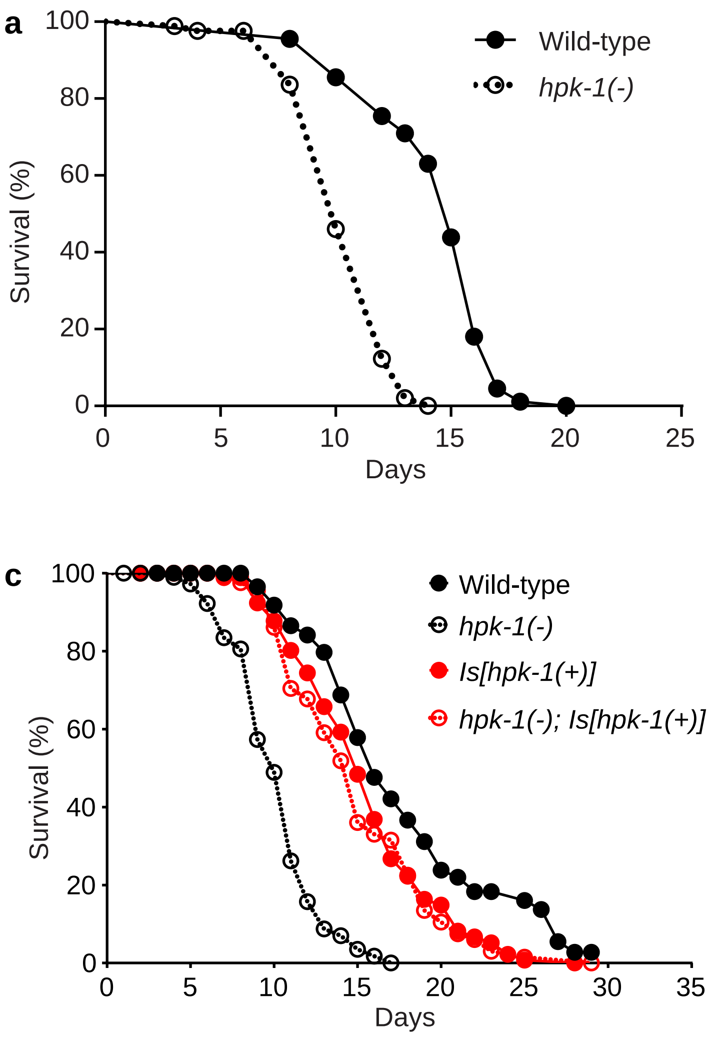
a
Wild-type
hpk-1(-)
Survival (%)
Days
b
Wild-type
hpk-1(-)
Survival (%)
Days
c
Wild-type
hpk-1(-)
Is[hpk-1(+)]
hpk-1(-); Is[hpk-1(+)]
Survival (%)
Days

a

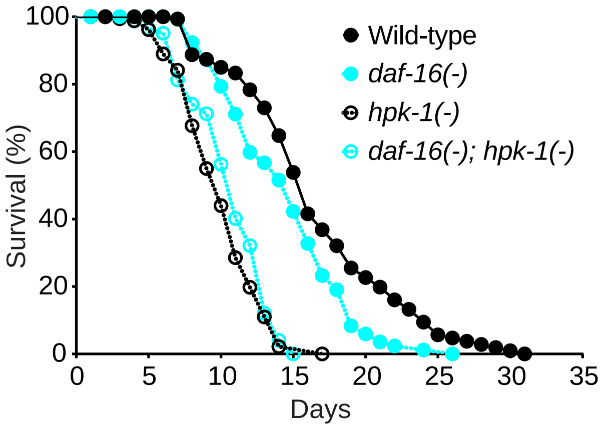
Wild-type
daf-16(-)
hpk-1(-)
daf-16(-); hpk-1(-)
Survival (%)
Days
0
20
40
60
80
100
0
5
10
15
20
25
30
35

Supplementary table 1. List of all the genes with decreased expression in *hpk-1(-)* animals vs. wild type animals by 1.5 fold or higher, p<0.05, listed in alphabetical order. Duplicates in red.

Gene	***hpk-1(-)*/ wild type**	**p-value**
C04E12.4	0.595	0.037
C06G8.2	0.597	0.032
C08B6.1	0.465	0.036
C14C6.5	0.148	0.014
C14C6.5	0.160	0.015
C27C12.1	0.584	0.029
C31E10.7	0.523	0.022
C36A4.1	0.346	0.018
C36A4.2	0.384	0.018
C36C5.5	0.481	0.046
C40H1.2	0.505	0.033
C41H7.3	0.607	0.048
C43H6.1	0.346	0.015
C43H6.1	0.459	0.021
C44C8.1	0.648	0.048
C48B4.1	0.409	0.035
C50H11.15	0.543	0.037
C52D10.1	0.406	0.015
C52D10.1	0.551	0.050
C54E4.5	0.500	0.031
C56A3.2	0.512	0.021
D1022.3	0.557	0.025
D1054.8	0.642	0.049
D1086.3	0.069	0.001
E01A2.7	0.553	0.042
F07A5.3	0.626	0.040
F08A8.2	0.346	0.012
F08H9.6	0.511	0.021
F08H9.6	0.488	0.022
F09F3.9	0.403	0.026
F10D2.9	0.140	0.028
F11A6.2	0.429	0.021
F14D7.6	0.559	0.043
F15E6.4	0.292	0.008
F17A2.13	0.620	0.050

F20D6.5	0.514	0.027
F25D1.5	0.525	0.042
F25G6.8	0.607	0.036
F31F4.15	0.319	0.020
F33C8.4	0.609	0.040
F35F10.13	0.580	0.047
F36D1.1	0.624	0.045
F38E11.1	0.471	0.016
F39E9.11	0.557	0.031
F39G3.1	0.597	0.047
F42A10.7	0.374	0.031
F42A8.1	0.413	0.014
F43C1.5	0.438	0.030
F44C4.3	0.318	0.015
F47G4.3	0.313	0.008
F47G9.3	0.486	0.031
F54F7.2	0.324	0.020
F56A8.1	0.521	0.028
F58A6.1	0.607	0.041
F58G6.2	0.516	0.022
H11E01.2	0.502	0.021
H13N06.6	0.553	0.025
H17B01.2	0.495	0.025
H17B01.3	0.345	0.019
H25K10.1	0.403	0.036
K01B6.3	0.620	0.045
K02B9.4	0.626	0.050
K03H1.6	0.540	0.025
K04G2.10	0.502	0.032
K07C6.5	0.261	0.033
K08C7.1	0.429	0.015
K09E2.3	0.410	0.014
K09E9.3	0.613	0.049
K10B3.9	0.622	0.044
K11C4.4	0.527	0.028
K11G9.5	0.574	0.033
K11G9.5	0.576	0.048
K11G9.6	0.299	0.011
M04C9.3	0.655	0.050
M142.1	0.646	0.048
M88.1	0.438	0.015

R03H10.6	0.333	0.015
R05D8.8	0.471	0.018
R07E5.4	0.434	0.041
R08H2.1	0.523	0.030
R09D1.11	0.549	0.030
R12A1.4	0.507	0.022
R12C12.9	0.595	0.041
T01G5.1	0.405	0.041
T01G5.7	0.561	0.048
T02B5.3	0.423	0.022
T03D3.1	0.361	0.022
T05E7.1	0.338	0.015
T06A1.5	0.622	0.040
T07D3.9	0.446	0.022
T08H10.1	0.580	0.042
T10G3.3	0.349	0.020
T14F9.3	0.568	0.047
T19H12.1	0.438	0.041
T22B7.7	0.228	0.038
T22F7.4	0.582	0.033
T24C2.2	0.622	0.050
T24C2.5	0.370	0.023
T28A11.2	0.588	0.034
W02H3.1	0.618	0.050
W06D12.5	0.588	0.031
W07B8.1	0.416	0.016
Y110A2AL.3	0.455	0.033
Y110A2AL.9	0.502	0.041
Y18D10A.25	0.511	0.045
Y34F4.2	0.622	0.044
Y38E10A.15	0.448	0.026
Y38E10A.4	0.365	0.050
Y45F10A.3	0.601	0.041
Y45G12C.2	0.396	0.020
Y46H3B.2	0.509	0.025
Y48E1B.8	0.163	0.018
Y4C6B.6	0.525	0.025
Y51H7C.1	0.420	0.042
Y54G11A.7	0.578	0.048
Y57G11B.5	0.545	0.048
Y69A2AR.12	0.639	0.044

Y6G8.2	0.503	0.048
Y71H2AL.1	0.639	0.047
Y73B6BL.25	0.624	0.043
Y73F4A.1	0.229	0.008
Y80D3A.9	0.622	0.048
ZC376.3	0.540	0.027
ZC412.3	0.648	0.049
ZC455.4	0.402	0.015
ZK185.3	0.455	0.027
ZK6.8	0.580	0.044
ZK792.2	0.620	0.039

Supplementary table 2. List of all the genes with increased expression in *hpk-1(-)* animals vs. wild type animals by 1.5 fold or higher, p<0.05, listed in alphabetical order. Duplicates in red.

Gene	*hpk-1(-)/* wild type	p-value
B0024.4	2.612	0.025
B0213.3	2.211	0.025
B0213.4	2.751	0.016
B0228.1	1.602	0.041
B0491.2	3.519	0.025
C01B4.6	3.138	0.020
C01B4.6	3.519	0.025
C01B4.8	3.182	0.015
C01B4.9	2.532	0.016
C02E7.6	4.042	0.018
C02F5.11	3.797	0.015
C02F5.8	2.211	0.018
C05A9.1	2.990	0.015
C05E7.1	1.741	0.037
C06A8.6	2.173	0.041
C07E3.4	1.753	0.043
C07G3.2	3.182	0.020
C09D4.3	2.282	0.033
C10C5.2	2.612	0.028
C15H7.3	2.445	0.039
C24F3.5	1.778	0.037
C24H11.1	1.670	0.048
C26B9.3	1.840	0.036
C30F2.3	1.693	0.039
C31H2.2	1.602	0.047
C33C12.4	2.329	0.030
C33G3.3	2.129	0.026
C33G8.4	1.847	0.048
C34D4.2	1.828	0.047
C34H4.2	2.338	0.046
C35B8.1	2.158	0.032
C35E7.9	2.657	0.025
C37C3.10	1.925	0.022
C41G7.6	2.071	0.048
C42D4.3	6.869	0.024
C43G2.3	1.979	0.033

C45B2.7	1.676	0.048
C49G7.10	2.938	0.016
C49G7.7	2.567	0.020
C50F2.10	3.364	0.022
C52E4.1	2.621	0.016
C54G4.3	2.014	0.041
D2096.6	1.803	0.034
EGAP9.2	1.699	0.031
F07C6.3	2.274	0.024
F08B1.1	1.853	0.047
F09F7.6	1.796	0.028
F10D11.6	2.035	0.023
F11G11.9	1.932	0.036
F14D7.7	2.227	0.022
F15B9.8	6.498	0.008
F20G2.5	2.121	0.050
F21C10.7	1.952	0.022
F21F3.3	2.959	0.024
F22H10.2	1.796	0.028
F23H12.4	4.317	0.016
F25E2.4	1.653	0.046
F27C1.8	6.453	0.014
F29B9.7	2.007	0.028
F31F6.4	2.181	0.021
F35B12.9	2.042	0.019
F35H10.4	1.653	0.041
F36A2.14	1.670	0.045
F36A4.2	2.196	0.015
F36A4.3	1.919	0.041
F36F12.7	1.821	0.025
F36H12.5	1.532	0.050
F37A8.1	1.784	0.033
F40F8.4	1.945	0.023
F41C3.2	5.169	0.036
F41F3.3	3.352	0.037
F42G8.8	1.608	0.041
F43C9.1	2.028	0.026
F44E2.4	1.912	0.033
F44F4.4	1.784	0.029
F44G4.5	2.000	0.025
F45D3.4	2.129	0.022

F45E4.5	1.790	0.026
F47D12.6	1.899	0.038
F47G6.4	1.613	0.041
F49F1.7	1.945	0.022
F52B10.1	2.329	0.015
F52B11.3	1.630	0.043
F53B2.5	2.621	0.031
F53F1.5	2.338	0.015
F55H12.1	1.899	0.036
F56A4.10	3.824	0.008
F56A4.12	3.127	0.015
F56A4.9	5.979	0.008
F56B3.6	1.608	0.040
F58A6.5	2.063	0.019
F58H1.2	1.866	0.032
F59D12.4	1.784	0.047
F59E12.9	1.840	0.048
H03A11.2	2.370	0.028
H05L14.1	1.932	0.050
H10E21.4	2.685	0.015
H23L24.5	2.488	0.013
H23N18.5	3.411	0.016
H36L18.2	1.558	0.050
K01D12.9	2.338	0.045
K01H12.2	1.821	0.041
K04F1.9	1.966	0.021
K05F1.9	2.532	0.021
K07A1.4	1.986	0.048
K07F5.6	1.853	0.033
K08H10.1	3.422	0.015
K08H10.1	3.317	0.032
K10D11.6	1.659	0.048
K11D12.4	4.042	0.008
K11D12.4	2.021	0.033
K11H12.4	2.462	0.014
M03A1.3	2.667	0.012
M03A1.7	4.377	0.015
R02E4.3	2.121	0.022
R06F6.11	1.711	0.036
R07C12.4	2.346	0.033
R08C7.8	2.021	0.032

R09B5.9	4.347	0.016
R09E10.3	2.056	0.040
R102.8	1.682	0.041
R12E2.15	3.364	0.031
R12E2.7	6.342	0.015
T02E1.6	1.723	0.048
T02E9.2	2.403	0.021
T05A10.3	2.585	0.020
T05A7.6	1.821	0.041
T10E9.6	1.682	0.045
T10E9.9	1.670	0.045
T18H9.1	2.979	0.023
T21E8.2	2.969	0.039
T22B2.6	1.630	0.048
T22C8.6	1.641	0.045
T24C4.4	3.758	0.008
W01D2.3	1.711	0.041
W01F3.2	1.765	0.026
W01F3.2	1.892	0.029
W01F3.3	2.085	0.049
W04G3.8	2.858	0.033
W06D4.2	1.866	0.036
W08E12.3	2.437	0.039
W08F4.6	2.648	0.036
Y105E8A.27	1.717	0.049
Y11D7A.9	2.166	0.033
Y19D10A.4	5.579	0.005
Y19D10A.4	5.370	0.008
Y19D10A.9	1.828	0.038
Y1A5A.1	2.488	0.032
Y37H2A.11	2.063	0.021
Y38C1BA.3	7.781	0.021
Y43C5B.3	1.778	0.032
Y46C8AL.8	1.723	0.048
Y46G5A.10	2.780	0.013
Y48B6A.5	1.847	0.050
Y50E8A.12	2.181	0.018
Y51A2D.11	1.741	0.050
Y51B9A.8	1.899	0.045
Y54E10BL.2	5.205	0.026
Y55B1BL.1	1.659	0.037

Y58A7A.3	2.630	0.023
Y65B4BR.1	2.558	0.049
Y73F8A.12	1.803	0.047
Y76B12C.2	1.972	0.047
ZK1010.7	5.483	0.016
ZK1025.2	2.742	0.025
ZK1025.3	3.877	0.015
ZK1025.6	3.063	0.015
ZK1067.7	2.362	0.044
ZK1290.8	1.853	0.032
ZK507.4	1.647	0.048
ZK822.4	1.717	0.030
ZK970.7	2.121	0.045

Supplementary table 3. Genes with decreased expression in *hpk-1(-)* animals vs. wild type animals and identified to be intestine enriched[35].

ID	Other gene name/function
F08H9.6	clec-57 (C-type LECtin)
R09D1.11	chil-23 / predicted to have chitinase activity
C36A4.1	cyp-25A1 (CYtochrome P450 family)
C36A4.2	cyp-25A2 (CYtochrome P450 family)
F09F3.9	cpt-5 (Carnitine Palmitoyl Transferase)
F31F4.15	fbxa-72 (F-box A protein)
F07A5.3	scav-6 (SCAVenger receptor (CD36 family) related)
F08A8.2	acox-2 (Acyl-Coenzyme A Oxidase)
F14D7.6	an ortholog of human MFSD8 (major facilitator superfamily domain containing 8)
F42A10.7	Uncharacterized protein
F10D2.9	fat-7 (FATty acid desaturase)
K11G9.6	mtl-1 (MeTaLlothionein)
F58G6.2	srm-3 (Serpentine Receptor, class M)
F25D1.5	Predicted to have oxidoreductase activity
F39G3.1	ugt-61 (UDP-GlucuronosylTransferase)
C40H1.2	Uncharacterized protein
Y6G8.2	non-coding

Supplementary table 4. Genes with increased expression in *hpk-1(-)* animals vs. wild type animals and identified to be intestine enriched[35].

ID	**Other gene name/function**
C07G3.2	irg-1 (Infection Response Gene)
C34H4.2	Uncharacterized protein
C49G7.10	Ortholog of human EPHX1 (epoxide hydrolase 1, microsomal (xenobiotic))
K11D12.4	cpt-4 (Carnitine Palmitoyl Transferase)
C52E4.1	CPROT/1; gcp-1; cpr-1 (Cysteine PRotease related)
F40F8.4	Uncharacterized protein
F43C9.1	Encodes a protein containing an F-box
H36L18.2	Uncharacterized protein
F25E2.4	ifd-2 (Intermediate Filament, D)
K10D11.6	Ortholog of human EPHX1 (epoxide hydrolase 1, microsomal (xenobiotic))
C02F5.8	tsp-1 (TetraSPanin family)
W01F3.2	Ortholog of human MMP19 (matrix metallopeptidase 19)
ZK822.4	Uncharacterized protein

Supplementary table 5. Genes with decreased expression in *hpk-1(-)* animals vs. wild type animals and identified to be age-regulated genes[38].

ID	gene name/function
F08H9.6#	clec-57 (C-type LECtin)
C14C6.5	Contains a Metridin-like ShK toxin domain
C36C5.5	Uncharacterized protein
C36A4.1#	cyp-25A1 (CYtochrome P450 family)
C36A4.2#	cyp-25A2 (CYtochrome P450 family)
F44C4.3	cpr-4 (Cysteine PRotease related)
D1086.3	Uncharacterized protein
F07A5.3#	scav-6 (SCAVenger receptor (CD36 family) related)
F15E6.4	Uncharacterized protein
F42A10.7#	Uncharacterized protein
F54F7.2	Uncharacterized protein
F10D2.9#	fat-7 (FATty acid desaturase)
K04G2.10	Uncharacterized protein
C48B4.1	Probable peroxisomal acyl-coenzyme A oxidase
T05E7.1	Predicted to have thiolester hydrolase activity
T07D3.9	Ortholog of human ACOT9 (acyl-CoA thioesterase 9)
T22B7.7	Ortholog of human ACOT9 (acyl-CoA thioesterase 9)
H13N06.6	tbh-1 (Tyramine Beta Hydroxylase)
ZC412.3	Uncharacterized protein ZC412.3
Y57G11B.5	Uncharacterized protein
D1054.8	Predicted to have oxidoreductase activity
H17B01.3	lips-14 /predicted to have hydrolase activity, based on protein domain

Intestine enriched genes

Supplementary table 6. Genes with increased expression in *hpk-1(-)* animals vs. wild type animals and identified to be age-regulated genes[38].

**ID**	**Other gene name/function**
C34H4.2#	Uncharacterized protein
ZK1010.7	col-97 (COLlagen)
C35B8.1	col-175 (COLlagen)
R09B5.9	cnc-4 (CaeNaCin (Caenorhabditis bacteriocin))
K11D12.4#	cpt-4 (Carnitine Palmitoyl Transferase)
C52E4.1#	CPROT/1; gcp-1; cpr-1 (Cysteine PRotease related)
C31H2.2	dpy-8 (DumPY : shorter than wild type)
F21F3.3	Predicted to have protein C-terminal S-isoprenylcysteine carboxyl O-methyltransferase activity
F40F8.4#	Uncharacterized protein
K10D11.6#	Ortholog of human EPHX1 (epoxide hydrolase 1, microsomal (xenobiotic)
B0213.4	nlp-29 (Neuropeptide-Like Protein)
ZK1025.6	nhr-244 (Nuclear Hormone Receptor family)
R09E10.3	acs-18 (fatty Acid CoA Synthetase family)
B0491.2	sqt-1 (SQuaT)
C02F5.8#	tsp-1 (TetraSPanin family)
W01F3.2#	an ortholog of human MMP19 (matrix metallopeptidase 19)
F08B1.1	vhp-1 (VH1 dual-specificity phosphatase family)

Intestine enriched genes

Supplementary table 7. Genes with decreased expression in *hpk-1(-)* animals vs. wild type animals and identified to be DAF-16 induced[40].

ID	Other gene name/function
C52D10.1	Uncharacterized protein
C56A3.2	ttr-44 (TransThyretin-Related family domain)
D1086.3	Uncharacterized protein
F10D2.9	fat-7 (FATty acid desaturase)
F15E6.4	Uncharacterized protein
F38E11.1	hsp-12.3 (Heat Shock Protein)
K11G9.6	met-I; mtl-1 (MeTaLlothionein)
R12A1.4	ges1; ges-1 (abnormal Gut ESterase)

Supplementary table 8. Genes with increased expression in *hpk-1(-)* animals vs. wild type animals and identified to be DAF-16 induced[40].

**ID**	**Other gene name/function**
C52E4.1#	CPROT/1; gcp-1; cpr-1 (Cysteine PRotease related)
F08B1.1	vhp-1; nrf-2, vhp-1 (VH1 dual-specificity phosphatase family)
F09F7.6	Uncharacterized protein
F21F3.3	Predicted to have protein C-terminal S-isoprenylcysteine carboxyl O-methyltransferase activity
F45D3.4	Uncharacterized protein
Y51A2D.11	ttr-26 (TransThyretin-Related family domain)
Y51B9A.8	Uncharacterized protein

[#] Intestine enriched genes

Supplementary table 9.

Tables indicate the p-value (to four decimal places) of all statistical testing conducted in this investigation. For more detail on statistical methods, please see the **Materials and methods** section.

Statistical Summary for Figure 1: HPK-1 levels are increased following heat shock

(C, E) Fluorescence intensity after heat stress – hpk-1::mCherry (Is[hpk1-1(+)]) *and* $_{P}$hpk-1::gfp

Unpaired t-test, two-tailed

**Comparison**	**p-value**
*Is[hpk-1(+)],*untreated vs. heat-shocked	0.0021
$_{P}$*hpk-1::gfp,* untreated vs. heat-shocked	0.2879

Statistical Summary for Figure 2: Loss of HPK-1 function confers sensitivity to heat stress.

(A) Heat stress

Two-way ANOVA, Sidak's multiple comparisons test (comparing heat-shocked data sets only)

**Comparison**	**p-value**
Wild-type vs. hpk-1(-)	< 0.0001
Wild-type vs. Is[hpk-1(+)]	0.5986
Wild-type vs. hpk-1; Is[hpk-1(+)]	> 0.9999

(B-D) Transcript levels of heat-shock proteins in wild type and hpk-1(-) *after heat-shock*

Two-way ANOVA, Sidak's multiple comparisons test (comparing wild-type and *hpk-1(-)* only)

	**Comparison (*Wild-type* vs. *hpk-1(-)*)**	
	Untreated	*Heat-shocked*
hsp-16.1/16.11	0.9899	0.9265
hsp-16.48/16.49	> 0.9999	0.7113
hsp-70	> 0.9999	0.5788

(F) Fluorescence intensity after heat-shock – $_{P}$hsp16.2::gfp

Two-way ANOVA, Sidak's multiple comparisons test

**Comparison**	**p-value**
Wild-type untreated vs. Wild-type heat-shocked	< 0.0001
Wild-type untreated vs. hpk-1(-) untreated	> 0.9999
hpk-1(-)untreated vs. hpk-1(-)heat-shocked	< 0.0001
Wild-type heat-shocked vs. hpk-1(-)heat-shocked	< 0.0001

Statistical Summary for Figure 3: Loss of HPK-1 function confers sensitivity to oxidative stress.

(A) Gamma irradiation

Two-way ANOVA, Sidak's multiple comparisons test

**Comparison**	**0 Gy**	**120 Gy**
Wild-type vs. hpk-1(-)	0.8737	< 0.0001
Wild-type vs. Is[hpk-1(+)]	> 0.9999	> 0.9999
Wild-type vs. hpk-1; Is[hpk-1(+)]	0.9976	0.0529

(B) Sodium Azide

Two-way ANOVA, Sidak's multiple comparisons test (Wild-type vs. *hpk-1(-)*)

**Concentration (mM)**	**p-value**
0	0.0425
7.8	< 0.0001
15.6	0.0001
31.25	< 0.0001
62.5	< 0.0001

Statistical Summary for Figure 4: Loss of HPK-1 function results in lifespan reduction and accelerated decline of physiological processes.

**Log-rank test (p-value)**	(A) Lifespan (including transgenic lines)	(B) Lifespan	(C) Fast body movement	(D) Pharyngeal pumping	(E) Fast pharyngeal pumping
Wild-type vs. hpk-1(-)	< 0.0001	< 0.0001	< 0.0001	< 0.0001	< 0.0001
Wild-type vs. Is[hpk-1(+)]	0.0040				
Wild-type vs. hpk-1(-); Is[hpk-1(+)]	0.2453				

**Median survival (days)**	(A) Lifespan (including transgenic lines)	(B) Lifespan	(C) Fast body movement	(D) Pharyngeal pumping	(E) Fast pharyngeal pumping
Wild-type	16	11	9	9	7
hpk-1(-)	10	9	5	7	5
Is[hpk-1(+)]	15				
hpk-1(-); Is[hpk-1(+)]	16				

Statistical Summary for Figure 5: HPK-1 involvement in the IIS pathway.

(A, B) sod-3 *and* ugt-9 *transcript levels in wild type and* hpk-1(-) *animals*

Unpaired t-test, two tailed

**Comparison (*Wild-type* vs *hpk-1(-)*)**	**p-value**
sod-3	0.0166
ugt-9	0.0199

(C) Lifespan

**Log-rank test (p-value)**	*Wild-type*	*daf-16(-)*	*hpk-1(-)*
daf-16(-)	< 0.0001		
hpk-1(-)	< 0.0001	< 0.0001	
daf-16(-); hpk-1(-)	< 0.0001	< 0.0001	0.2624
**Median survival (days)**			
Wild-type	17		
daf-16(-)	15		
hpk-1(-)	11		
daf-16(-); hpk-1(-)	11		

Statistical Summary for Supplementary Figure S1: Loss of HPK-1 does not affect DNA damage response in the germline after gamma irradiation.

(B) Cell cycle arrest following gamma irradiation

Two-way ANOVA, Sidak's multiple comparisons test

**Comparison (0 Gy vs. 120 Gy)**	**p-value**
Wild-type	0.0046
hpk-1(-)	0.0117

(D) Number of apoptotic corpses following gamma irradiation

Two-way ANOVA, Sidak's multiple comparisons test

**Comparison (0 Gy vs. 120 Gy)**	**p-value**
Wild-type	0.0390
hpk-1(-)	0.0451

Statistical Summary for Supplementary Figure S2: Loss of HPK-1 function results in a lifespan reduction.

Replicate lifespan assays for Figure 4

**Log-rank test (p-value)**	(A) Lifespan	(B) Lifespan	(C) Lifespan
Wild-type vs. hpk-1(-)	< 0.0001	< 0.0001	< 0.0001
Wild-type vs. Is[hpk-1(+)]			0.0016
Wild-type vs. hpk-1(-); Is[hpk-1(+)]			0.0014
**Median survival (days)**	(A) Lifespan	(B) Lifespan	(C) Lifespan
Wild-type	15	17	16
hpk-1(-)	10	9	10
Is[hpk-1(+)]			15
hpk-1(-); Is[hpk-1(+)]			15

Statistical Summary for Supplementary Figure S3: HPK-1 involvement in the IIS Pathway.

Replicate lifespan assay for Figure 5

**Log-rank test (p-value)**	*Wild-type*	*daf-16(-)*	*hpk-1(-)*
daf-16(-)	0.0012		
hpk-1(-)	< 0.0001	< 0.0001	
daf-16(-); hpk-1(-)	< 0.0001	< 0.0001	0.2847
**Median survival (days)**			
Wild-type	16		
daf-16(-)	15		
hpk-1(-)	10		
daf-16(-); hpk-1(-)	11		

Supplementary methods

Cell cycle arrest

Young adults were treated with 120 Gy of gamma irradiation using a caesium 137 source. Gonads were extruded from animals in a drop of 0.001% (w/v) tetramisole on a cover slip using 0.60x32 mm needles followed by placement on poly-L-lysine coated slide by inverting. These were then frozen in liquid nitrogen followed by removal of the cover slip i.e. freeze-cracked. Fixing was performed in 100% methanol (high purity grade and previously cooled at -20 °C) for 30-60 seconds followed by 30 min incubation in 1xPBS, 0.08 M Hepes (pH 6.9), 1.6 mM MgSO4, 0.8 mM EGTA, 3.7% paraformaldehyde. Slides were then washed twice in 1x PBST (1xPBS, 0.5% Tween 20) for 10 minutes each time. Following washing in PBST, a drop of mounting media containing DAPI (1.5 µg/ml) was added to each slide and the cover slip placed over. Imaging was performed once the mounting media had set (usually after a day or two). Images of a single plane of mitotic regions were taken using BX51 Microscope (Olympus). Scoring of the number of cells within the single plane of view was performed using Image J software.

Acridine orange staining

Young adults were treated with 120 Gy of gamma irradiation using a caesium 137 source. 24 hours after the treatment, 1 ml of acridine orange solution in M9 buffer (50 µg/ml) was added to 60 mm NGM plates on which worms were grown. The solution was distributed evenly over the agar surface by gentle tilting of the plates. These were then left in the dark for one hour after which the animals were washed in M9 buffer three times by allowing pellets to form. Washed worms were plated out on to fresh OP50 seeded NGM plates and imaged after an hour with DIC and fluorescence optics using BX51 Microscope (Olympus)
